# mTOR pathway gene expression in association with race and clinicopathological characteristics in Black and White breast cancer patients

**DOI:** 10.1007/s12672-022-00497-y

**Published:** 2022-05-24

**Authors:** Mmadili N. Ilozumba, Song Yao, Adana A. M. Llanos, Angela R. Omilian, Weizhou Zhang, Susmita Datta, Chi-Chen Hong, Warren Davis, Thaer Khoury, Elisa V. Bandera, Michael Higgins, Christine B. Ambrosone, Ting-Yuan David Cheng

**Affiliations:** 1 Department of Epidemiology, University of Florida, Gainesville, FL USA; 2 Department of Cancer Prevention and Control, Roswell Park Comprehensive Cancer Center, Buffalo, NY USA; 3 Department of Epidemiology, Mailman School of Public Health and Herbert Irving Comprehensive Cancer Center, Columbia University Irving Medical Center, NY New York, United States; 4 Department of Pathology, Immunology and Laboratory Medicine, University of Florida, Gainesville, FL USA; 5 Department of Biostatistics, University of Florida, Gainesville, FL USA; 6 Department of Pathology & Laboratory Medicine, Roswell Park Comprehensive Cancer Center, Buffalo, NY USA; 7 Cancer Epidemiology and Health Outcomes, Rutgers Cancer Institute of New Jersey, New Brunswick, NJ United States; 8 Department of Molecular and Cellular Biology, Roswell Park Comprehensive Cancer Center, Buffalo, NY USA; 9 Division of Cancer Prevention and Control, Department of Internal Medicine, The Ohio State University, Suite 525, 1590 North High Street, Columbus, OH 43201 USA

**Keywords:** mTOR, Gene expression, Breast cancer, Race, Clinicopathological characteristics

## Abstract

**Background:**

Aberrant activation of the mammalian Target of Rapamycin (mTOR) pathway has been linked to obesity and endocrine therapy resistance, factors that may contribute to Black-White disparities in breast cancer outcomes. We evaluated associations of race and clinicopathological characteristics with mRNA expression of key mTOR pathway genes in breast tumors.

**Methods:**

Surgical tumor tissue blocks were collected from 367 newly diagnosed breast cancer patients (190 Black and 177 White). Gene expression of *AKT1*, *EIF4EBP1*, *MTOR*, *RPS6KB2*, and *TSC1* were quantified by NanoString nCounter. Differential gene expression was assessed using linear regression on log2-transformed values. Gene expression and DNA methylation data from TCGA were used for validation and investigation of race-related differences.

**Results:**

Compared to White women, Black women had relative under-expression of *AKT1* (log2 fold-change = − 0.31, 95% CI − 0.44, − 0.18) and *RPS6KB2* (log2 fold-change = **− **0.11, 95% CI − 0.19, − 0.03). Higher vs. lower tumor grade was associated with relative over-expression of *EIF4EBP1* and *RPS6KB2*, but with lower expression of *TSC1*. Compared to luminal tumors, triple-negative tumors had relative under-expression of *TSC1* (log2 fold-change = − 0.42, 95% CI − 0.22, − 0.01). The results were similar in the TCGA breast cancer dataset. Post-hoc analyses identified differential CpG methylation within the *AKT1* and *RPS6KB2* locus between Black and White women.

**Conclusions:**

Over-expression of *RPS6KB2* and *EIF4EBP1* and under-expression of *TSC1* might be indicators of more aggressive breast cancer phenotypes. Differential expression of *AKT1* and *RPS6KB2* by race warrants further investigation to elucidate their roles in racial disparities of treatment resistance and outcomes between Black and White women with breast cancer.

**Supplementary information:**

The online version contains supplementary material available at 10.1007/s12672-022-00497-y.

## Introduction

Breast cancer mortality is at least 40% higher in Black women compared to White women in the US [1]. The reason for this disparity gap is complex and likely involves both social and biological determinants. Breast tumors with more aggressive phenotypes, including higher grade, hormone receptor (HR)-negative, and triple-negative breast cancer (TNBC) subtype, are more common in Black women than in White women with breast cancer [2]. Obesity, an important prognostic factor of breast cancer [3, 4], likely contributes to the disparities in breast cancer mortality between Black and White women as obesity is more prevalent in Black women compared to White women [5]. In addition, Black women with HR-positive tumors have higher risks of recurrence and mortality relative to White women with the same breast cancer subtype [6, 7], which might be due to differences in resistance to endocrine therapy. Improving our understanding of biomarkers and molecular mechanisms involved in obesity-signaling pathways and endocrine therapy resistance and potentially different by race is important for planning mechanism-based therapeutic interventions to close the disparity gap in breast cancer mortality.

**Fig. 1:**
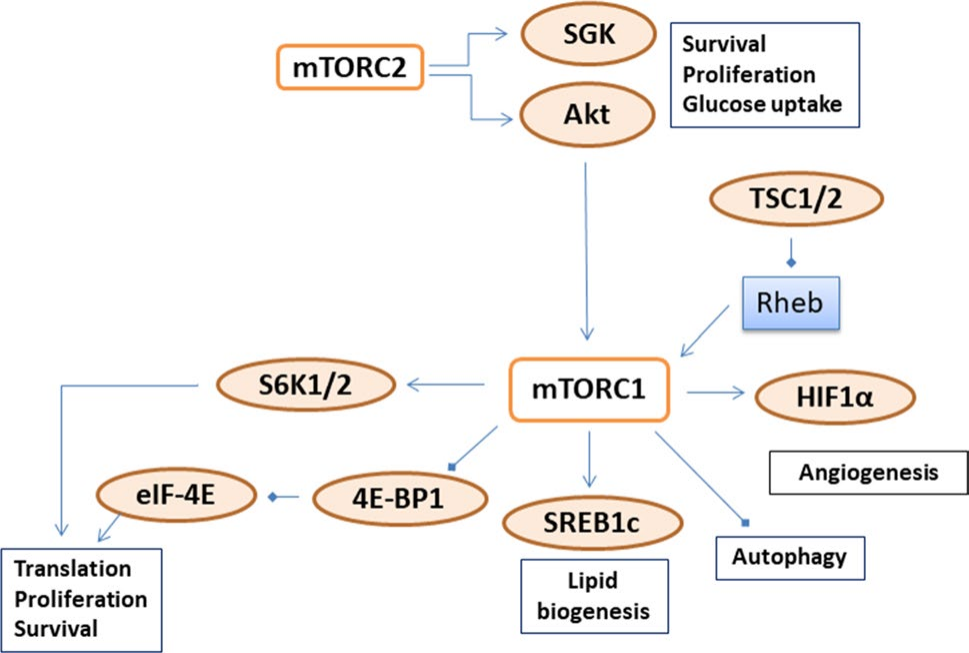
mTOR signaling pathway in cancer (revised from Zoncu et al. Nature Reviews. 2011 12:21–35). *4E-BP1* eukaryotic translation initiation factor 4E-binding protein 1; *Akt* protein kinase B; *HIF1α* hypoxia-inducible factor 1α; *eIF-4E* eukaryotic translation initiation factor 4E; *mTORC1* mammalian Target of Rapamycin complex 1; *mTORC2* mammalian Target of Rapamycin complex 2; *Rheb* Ras Homologue enriched in brain; *S6K1/2* p70 ribosomal S6 kinase 1 and 2; *SGK* serum- and glucocorticoid-regulated kinase; *SREBP1* sterol regulatory element binding protein 1c; *TSC1/2* tuberous sclerosis protein 1 and 2

Genes in the phosphatidylinositol 3-kinase/AKT/mammalian target of rapamycin (mTOR) pathway are highly expressed in breast cancer and are related to prognosis [8–13]. Specifically, mTOR, which forms mTOR complex 1 (mTORC1) and mTOR complex 2 (mTORC2), functions as a serine/threonine protein kinase that regulates protein synthesis, proliferation, and autophagy [14, 15] (Fig. 1). mTORC1 regulates several proteins, including the p70 ribosomal S6 kinases (S6K1 and S6K2), which directly phosphorylate the activation domain of the estrogen receptor (ER), a mechanism for resistance to endocrine therapy in ER + tumors [16–19]. mTORC1 also regulates protein synthesis through inhibiting 4E-binding protein-1 (4E-BP1) and eukaryotic initiation factor 4E (eIF-4E) [20–22]. In addition, mTORC1 is negatively regulated by tuberous sclerosis protein 1 and 2 (TSC1–TSC2) complex [23, 24]. Loss of *TSC1* or *TSC2* expression has been shown to activate mTORC1 and its downstream factors [23, 24].

The mTOR pathway can be promoted by positive energy imbalance, insulin resistance, and elevated insulin-like growth factors (IGFs) [25, 26]. Black women may be predisposed to higher mTOR activation as they have higher prevalence of obesity, insulin resistance, and diabetes compared to White women [13, 27]. Our recent data suggest that Black women have higher protein expression and activation levels of mTOR in their breast tumors than White women [13]. In addition, we found that higher levels of pathway activity—indicated by the expression of phosphorylated (p)-mTOR, p-AKT, and p-S6K1—were associated with lower tumor grade, smaller tumors, earlier stage at diagnosis, and luminal subtype. However, it remains unclear to what extent the expression levels of these genes differ by race and clinicopathological characteristics.

To address this gap in knowledge, we examined associations of race and breast tumor clinicopathological characteristics with the expression of key genes in the mTOR pathway, including *AKT1*, *MTOR*, *RPS6KB2* (encodes S6K2), *EIF4EBP1* (encodes eIF-4E), and *TSC1*, in the mTOR pathway in Black and White women with newly diagnosed breast cancer. We hypothesized that the gene expression levels of *AKT1*, *MTOR*, and *RPS6KB2* are higher in tumors from Black women compared to White women, but expression of *EIF4EBP1* and *TSC1*, two genes that may have lower expression when mTOR is overexpressed, are lower among Black women compared to White women. We also hypothesized that gene expression is higher in tumors with features indicative of more aggressive phenotype, including higher grade and ER- or TNBC subtypes, relative to less aggressive features. For validation purposes, we also examined gene expression and DNA methylation data from The Cancer Genome Atlas (TCGA).

## Methods

### Study population

Patients were selected from the Women’s Circle of Health Study (WCHS). Black patients were frequency matched to White patients by age at diagnosis and tumor subtype. To increase the representation of HER2 and triple-negative subtypes, which are less common than luminal subtype, we over-sampled HER2 and triple-negative subtypes for both Black and White groups. Because WCHS did not have enough cancer cases needed for the matching and oversampling design, we added patients from Pathology Network Shared Resources (PNSR) at the Roswell Park Comprehensive Cancer Center. As a result, 190 Black patients and matched 177 White patients were selected (total N = 367). The design and data and biospecimen collection in WCHS have been described elsewhere [13, 28, 29]. Questionnaire data on known and suspected risk factors for breast cancer and anthropometric measurements were obtained during in-person interviews. Clinical and tumor characteristics and receptor status were obtained from pathology reports.

### Tumor tissue processing and gene expression profiling

Formalin-fixed paraffin-embedded (FFPE) tumor tissue specimens were used for RNA extraction. Two 10 μm curls were cut from a FFPE block and RNA was extracted using the High Pure FFPET RNA Isolation Kit (Roche Molecular Systems, Inc., Pleasanton, CA, USA) and quantified using Qubit and Agilent Bioanalyzer (Agilent Technologies, Santa Clara, CA, USA). *AKT1*, *EIF4EBP1*, *MTOR*, *RPS6KB2*, *TSC1* were added as custom content to the NanoString PanCancer Immune Panel and quantitated using NanoString nCounter^®^ technology (NanoString Technologies, Seattle, WA, USA). Samples included 15 technical duplicates and one biological trio (from three different tumor blocks of the same case). All samples passed the QC measures following manufacturer’s recommendations. Among the panel, the correlations of the gene counts were in the range of 0.98–0.99, with one exception of an R = 0.89, between each of the technical duplicative pairs. The correlations of the gene counts among the biological trio samples ranged from 0.98 to 0.99. In addition, DNA methylation data generated by Illumina 450 K BeadChip were available for 54 WCHS participants to examine the correlations between gene expression and DNA methylation [30].

### Statistical analysis

Gene expression data were first normalized to positive controls included in the assays and then a panel of 40 house-keeping genes using geNorm algorithm, and the data were log2-transformed. Pairwise correlations of gene expression levels were assessed using Pearson correlation analysis. Multivariable linear regression analyses were performed to assess the log2 fold-change of gene expression across race and clinicopathological characteristics. Models included race (White, Black), age (≤ 40, 41–50, 51–65, > 65), tumor grade (low, intermediate, high), ER status (negative, positive), progesterone receptor (PR) status (negative, positive), HER2 status (negative, positive), and three-marker molecular subtype (Luminal: ER+/PR+/HER2 + or ER+/PR+/HER2-, HER2-overexpressed: ER-/PR-/HER2+, and triple-negative: ER-/PR-/HER2-). Because approximately 10% of the patients did not have data on breast cancer stage, we included the variable in separate models as a sensitivity analysis. Because Black women have a higher prevalence of obesity (BMI ≥ 30) than White women, and BMI is associated with mTOR activation [13], we conducted subgroup analysis additionally adjusting for BMI in 150 WCHS participants with available data. All statistical tests were 2-sided, and p-value < 0.05 was considered statistically significant. Analyses were performed using SAS version 9.3 (SAS Institute Inc.).

### External validation of mTOR gene expression and DNA methylation  analysis using data from The Cancer Genome Atlas (TCGA)

We downloaded GDC TCGA Breast cancer (BRCA) dataset from the University of California Santa Cruz (UCSC) Xena Portal (http://xena.ucsc.edu). We selected genomic data on *AKT1*, *EIF4EBP1*, *MTOR*, *RPS6KB2*, *TSC1*, as well as phenotypic data on race and age. We merged the gene expression data with the clinicopathologic data, including ER, PR, and HER2 status, PAM 50 intrinsic subtype annotation, and tumor stage classified by the American Joint Committee on Cancer (AJCC), from TCGA [8]. A total of 332 (30 Black and 302 White) breast cancer patients were included in the analysis. Since the number of Black women was low, the TCGA data were primarily used to validate our findings of gene expression associations with tumor characteristics in WCHS.

Given that DNA methylation may downregulate gene expression, a post-hoc analysis was conducted to explore differences between CpG site-specific methylation for *AKT1* and *RPS6KB2* by race using TCGA’s Illumina Infinium Human Methylation 450k array dataset from UCSC Xena portal. CpG island (CGI) positions were identified based on 1500 bp genomic length scale. Differences in DNA methylation (β), i.e., the ratio of methylation levels between methylated and unmethylated alleles, between 164 Black women and 670 White women were examined using one-way ANOVA; p-values were corrected for multiple comparisons using the False Discovery Rate (FDR) method at 0.05 level. We further determined the gene-level correlation of methylation level at CGI loci and gene expression using Pearson correlation. The DNA methylation analyses were performed using the SMART (Shiny Methylation Analysis Resource Tool) App.

## Results

Table 1 shows the characteristics of the study participants overall and by race. Almost two-thirds (65.83%) had high-grade tumors; 50.4% were luminal tumors, 12.5% were HER2-overexpressed, and 37.1% were TNBC.

**Table 1:** Characteristics of the study participants

Characteristic	Overall N = 367	White n = 177	Black n = 190
Age, mean (SD)	54.07 (11.99)	54.76 (13.38)	53.43 (10.52)
Age, n (%)			
≤ 40	47 (12.81)	30 (16.95)	17 (8.95)
41–50	103 (28.07)	43 (24.29)	60 (31.58)
51–65	156 (42.51)	65 (36.72)	91 (47.89)
> 65	61 (16.62)	39 (22.03)	22 (11.58)
BMI, mean (SD)^a^	30.93 (7.49)	26.90 (6.69)	32.08 (7.33)
BMI, n (%)			
< 25	35 (22.88)	16 (47.06)	19 (15.97)
25–< 30	42 (27.45)	7 (20.59)	35 (29.41)
≥ 30	76 (49.67)	11 (32.35)	65 (54.62)
Tumor Grade, n (%)			
Low	26 (7.22)	15 (8.57)	11 (5.95)
Intermediate	97 (26.94)	46 (26.29)	51 (27.57)
High	237 (65.83)	114 (65.14)	123 (66.49)
Missing	7 (1.91)	2 (1.13)	5 (2.63)
AJCC stage			
Stage I	181 (55.69)	93 (58.86)	88 (52.69)
Stage II	122 (37.54)	56 (35.44)	66 (39.52)
Stage III	19 (5.85)	7 (4.43)	12 (7.19)
Stage IV	3 (0.92)	2 (1.27)	1 (0.60)
Missing	40 (10.96)	17 (9.71)	23 (12.11)
ER status, n (%)			
Positive	181 (49.32)	91 (51.41)	90 (47.37)
Negative	186 (50.68)	86 (48.59)	100 (52.63)
PR status, n (%)			
Positive	154 (41.96)	74 (41.81)	80 (42.11)
Negative	213 (58.04)	103 (58.19)	110 (57.89)
HER2 status, n (%)			
Negative	235 (64.03)	105 (59.32)	130 (68.42)
Positive	132 (35.97)	72 (40.68)	60 (31.58)
Molecular subtype, n (%)			
Luminal	185 (50.41)	92 (51.98)	93 (48.95)
HER2+	46 (12.53)	20 (11.30)	26 (13.68)
Triple-negative	136 (37.06)	65 (36.72)	71 (37.37)

^a^Data on BMI were available among 119 Black patients and 34 White patients in WCHS

Pearson’s correlation coefficients among the mTOR pathway gene expressions overall and separately among Black women and White women are presented in Supplemental Table 1. Overall, there were weak positive correlations for *AKT1* with *MTOR* and *TSC1* (r = 0.17 and 0.19, respectively), but a moderate positive correlation with *RPS6KB2* (r = 0.46). There was a moderate positive correlation for *EIF4EBP1* with *RPS6KB2* (r = 0.30) and a moderate negative correlation with *TSC1* (− 0.42). There were weak positive correlations for *MTOR* with *RPS6KB2* and *TSC1* (r = 0.18 and 0.12, respectively). These remained consistent in the race-specific analysis.

**Fig. 2:**
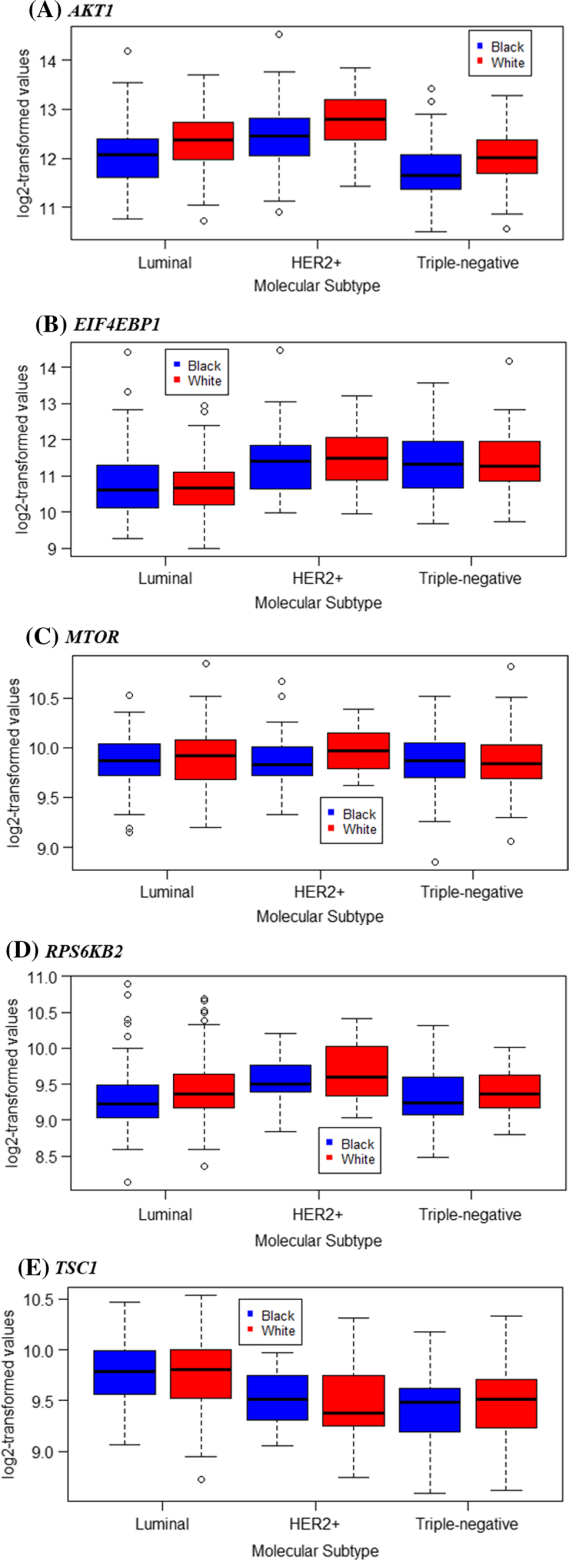
mTOR gene expression levels among Black women vs. White women, stratified by molecular subtypes. **A** Luminal, p-value = 0.0003; HER2+, p-value = 0.1696; Triple-negative, p-value = 0.0028, **B** Luminal, p-value = 0.8275; HER2+, p-value = 0.8599; Triple-negative, p-value = 0.9744, **C** Luminal, p-value = 0.7066; HER2+, p-value = 0.3314; Triple-negative, p-value = 0.8613, **D** Luminal, p-value = 0.0348; HER2+, p-value = 0.3720; Triple-negative, p-value = 0.1132, **E** Luminal, p-value = 0.7842; HER2+, p-value = 0.6807; Triple-negative, p-value = 0.3711

Table 2 shows means and standard deviations of log2-transformed gene expression levels by race and clinicopathological characteristics. Tumors from Black women had significantly lower *AKT1* and *RPS6KB2* expression compared to those from White women. By molecular subtype, the racial difference was observed in luminal and TNBC tumors for *AKT1* (Fig. 2A) and luminal tumors for *RPS6KB2* (Fig. 2D). Tumors with higher grade had higher expression of *EIF4EBP1* and *RPS6KB2* but lower expression of *TSC1* relative to tumors with lower grade. ER + tumors had higher expression levels of *AKT1* and *TSC1* but lower expression levels of *EIF4EBP1* compared to ER- tumors. HER2 + tumors had higher expression of *AKT1* and *RPS6KB2* compared to HER2- tumors. Among the molecular subtypes, HER2-overexpressed tumors had the highest expression of *AKT1*, *EIF4EBP1* and *RPS6KB2*.

**Table 2:** Gene expression levels (log2-transformed values) of the mTOR pathway in breast cancer according to race, age, and clinicopathological characteristics (N = 367)

		*AKT1*		*EIF4EBP1*		*MTOR*		*RPS6KB2*		*TSC1*	
Characteristic	N	Mean ± SD	p-value	Mean ± SD	p-value	Mean ± SD	p-value	Mean ± SD	p-value	Mean ± SD	p-value
Race			** < 0.0001**		0.7645		0.6702		**0.0095**		0.6679
White	177	12.31 ± 0.63		11.04 ± 0.86		9.88 ± 0.30		9.45 ± 0.40		9.63 ± 0.38	
Black	190	12.00 ± 0.66		11.07 ± 0.97		9.87 ± 0.28		9.34 ± 0.40		9.62 ± 0.35	
Age			0.7837		0.9160		0.4335		0.2188		
≤ 40	47	12.21 ± 0.58		11.12 ± 0.80		9.86 ± 0.30		9.50 ± 0.42		9.54 ± 0.35	0.2829
41—50	103	12.18 ± 0.66		11.06 ± 1.02		9.90 ± 0.30		9.39 ± 0.44		9.66 ± 0.40	
51—65	156	12.13 ± 0.72		11.06 ± 0.93		9.85 ± 0.29		9.36 ± 0.39		9.63 ± 0.34	
> 65	61	12.11 ± 0.58		10.99 ± 0.81		9.89 ± 0.26		9.39 ± 0.37		9.62 ± 0.36	
Tumor grade			0.4616		** < 0.0001**		0.6102		** < 0.0001**		** < 0.0001**
Low	26	12.03 ± 0.47		10.18 ± 0.50		9.92 ± 0.22		9.10 ± 0.21		9.92 ± 0.23	
Intermediate	97	12.20 ± 0.61		10.72 ± 0.80		9.87 ± 0.29		9.31 ± 0.42		9.80 ± 0.32	
High	237	12.14 ± 0.71		11.30 ± 0.88		9.86 ± 0.30		9.46 ± 0.40		9.52 ± 0.35	
ER status			0.0044		** < 0.0001**		0.8478		0.3512		** < 0.0001**
Positive	181	12.25 ± 0.63		10.75 ± 0.86		9.87 ± 0.28		9.37 ± 0.44		9.76 ± 0.34	
Negative	186	12.05 ± 0.68		11.35 ± 0.88		9.87 ± 0.30		9.41 ± 0.36		9.49 ± 0.35	
PR status			0.1461		** < 0.0001**		0.7403		0.0795		** < 0.0001**
Positive	154	12.21 ± 0.59		10.71 ± 0.86		9.88 ± 0.28		9.35 ± 0.42		9.76 ± 0.33	
Negative	213	12.11 ± 0.71		11.30 ± 0.88		9.87 ± 0.30		9.42 ± 0.39		9.53 ± 0.36	
HER2 status			** < 0.0001**		0.7117		0.7186		** < 0.0001**		0.9911
Negative	235	11.97 ± 0.58		11.04 ± 0.92		9.88 ± 0.29		9.31 ± 0.38		9.63 ± 0.38	
Positive	132	12.47 ± 0.67		11.08 ± 0.92		9.86 ± 0.28		9.53 ± 0.40		9.63 ± 0.34	
Molecular subtype			** < 0.0001**		** < 0.0001**		0.3447		**0.0002**		** < 0.0001**
Luminal	185	12.25 ± 0.62		10.75 ± 0.85		9.87 ± 0.28		9.37 ± 0.44		9.76 ± 0.33	
HER2 +	46	12.58 ± 0.73		11.40 ± 0.96		9.93 ± 0.27		9.62 ± 0.35		9.52 ± 0.31	
Triple-negative	136	11.88 ± 0.57		11.35 ± 0.86		9.86 ± 0.31		9.35 ± 0.34		9.47 ± 0.36	

Bolded values indicate statistically significant results with p < 0.05

In multivariable models (Table 3), Black women had relative under-expression of *AKT1* (log2 fold-change = − 0.31, 95% CI − 0.44, -0.18) and *RPS6KB2* (log2 fold-change = − 0.11, 95% CI − 0.19, − 0.03) in their breast tumors compared to White women. High vs. low tumor grade had relative over-expression of *EIF4EBP1* (log2 fold-change = 0.84, 95% CI 0.46, 1.22) and *RPS6KB2* (log2 fold-change = 0.33, 95% CI 0.15, 0.50), but under-expression of *TSC1* (log2 fold-change = − 0.23, 95% CI − 0.38, − 0.08). HER2 + vs. HER2- status was associated with relative over-expression of *AKT1* (log2 fold-change = 0.23, 95% CI 0.04, 0.42) and *RPS6KB2* (log2 fold-change = 0.13, 95% CI 0.01, 0.25), but relative under-expression of *TSC1* (log2 fold-change = − 0.12, 95% CI − 0.22, − 0.01). In comparison to luminal tumors, TNBC subtype had lower expression of *TSC1* (log2 fold-change = − 0.42, 95% CI − 0.77, − 0.07). These associations remained similar after additionally adjusted for breast cancer stage (Supplemental Table 2), despite that a more advanced stage was associated with relative over-expression of *AKT1* and *RPS6KB2*, compared to an earlier stage. Among patients with data on BMI, the subgroup analysis additionally adjusting for BMI showed consistent results (Supplemental Table 3).

**Table 3:** Association between race and clinicopathological characteristics and gene expression of the mTOR pathway in breast cancer (n = 360)^a^

	*AKT1*		*EIF4EBP1*		*MTOR*		*RPS6KB2*		*TSC1*	
Characteristic	Log2 fold-change (95% CI)	p-value	Log2 fold-change (95% CI)	p-value	Log2 fold-change (95% CI)	p-value	Log2 fold-change (95% CI)	p-value	Log2 fold-change (95% CI)	p-value
Race										
White	Ref.		Ref.		Ref.		Ref.		Ref.	
Black	− 0.31 (− 0.44, − 0.18)	**< 0.0001**	− 0.02 (− 0.20, 0.16)	0.8320	− 0.02 (− 0.08, 0.04)	0.5001	− 0.11 (− 0.19, − 0.03)	**0.0105**	− 0.02 (− 0.09, 0.05)	0.5891
Age										
≤ 40	Ref.		Ref.		Ref.		Ref.		Ref.	
41–50	0.06 (− 0.15, 0.27)	0.5850	0.03 (− 0.26, 0.33)	0.8208	0.04 (− 0.06, 0.14)	0.4647	− 0.05 (− 0.18, 0.08)	0.4651	0.08 (− 0.03, 0.20)	0.1589
51–65	− 0.01 (− 0.21, 0.19)	0.9252	0.05 (− 0.22, 0.33)	0.6959	− 0.02 (− 0.11, 0.08)	0.7367	− 0.08 (− 0.21, 0.04)	0.1869	0.05 (− 0.06, 0.16)	0.3678
> 65	− 0.14 (− 0.37, 0.10)	0.2481	− 0.13 (− 0.45, 0.19)	0.4360	0.03 (− 0.08, 0.14)	0.5982	− 0.11 (− 0.26, 0.03)	0.1293	0.08 (− 0.05, 0.21)	0.2079
Tumor grade										
Low	Ref.		Ref.		Ref.		Ref.		Ref.	
Intermediate	0.18 (− 0.09, 0.45)	0.2024	0.43 (0.06, 0.81)	**0.0243**	− 0.03 (− 0.16, 0.10)	0.6809	0.19 (0.01, 0.36)	**0.0332**	− 0.04 (− 0.18, 0.11)	0.6229
High	0.16 (− 0.11, 0.43)	0.2494	0.84 (0.46, 1.22)	**< 0.0001**	− 0.03 (-0.17, 0.10)	0.6104	0.33 (0.15, 0.50)	**0.0002**	− 0.23 (-0.38, -0.08)	**0.0024**
ER status										
Positive	Ref.		Ref.		Ref.		Ref.		Ref.	
Negative	0.002 (− 0.61, 0.61)	0.9950	− 0.13 (− 0.98, 0.71)	0.7556	− 0.05 (− 0.35, 0.25)	0.7442	− 0.06 (− 0.45, 0.32)	0.7435	0.10 (− 0.23, 0.43)	0.5607
PR status										
Positive	Ref.		Ref.		Ref.		Ref.		Ref.	
Negative	0.20 (− 0.04, 0.44)	0.0970	0.09 (− 0.24, 0.42)	0.5917	− 0.04 (− 0.16, 0.07)	0.4701	0.08 (− 0.07, 0.23)	0.3074	0.07 (− 0.06, 0.20)	0.2746
HER2 status										
Negative	Ref.		Ref.		Ref.		Ref.		Ref.	
Positive	0.23 (0.04, 0.42)	**0.0172**	0.13 (− 0.13, 0.40)	0.3229	− 0.07 (− 0.17, 0.02)	0.1229	0.13 (0.01, 0.25)	**0.0338**	− 0.12 (− 0.22, − 0.01)	**0.0289**
Molecular subtype										
Luminal	Ref.		Ref.		Ref.		Ref.		Ref.	
HER2+	0.07 (− 0.61, 0.76)	0.8390	0.40 (-0.55, 1.35)	0.4080	0.18 (− 0.15, 0.52)	0.2789	0.09 (− 0.34, 0.53)	0.6689	− 0.26 (− 0.64, 0.11)	0.1625
Triple-negative	− 0.43 (− 1.08, 0.21)	0.1879	0.49 (-0.41, 1.38)	0.2847	0.05 (− 0.27, 0.36)	0.7712	− 0.05 (− 0.45, 0.36)	0.8274	− 0.42 (− 0.77, − 0.07)	**0.0197**

*CI* confidence interval; Bolded values indicate statistically significant results with p < 0.05

^a﻿^Linear regression models included race, age, tumor grade, ER status, PR status, HER2 status, and molecular subtype

### External validation in the TCGA breast cancer dataset and the post-hoc analysis of DNA methylation data

Participant characteristics and the results of the analysis of TCGA data are shown in Supplemental Tables 4–6. Compared to luminal tumors, *TSC1* gene expression was relatively under-expressed in HER2-enriched tumors and Basal-like tumors (Supplemental Table 6). These findings were consistent with our main analysis in WCHS. Additionally, *EIF4EBP1* gene expression was relatively over-expressed in HER2-enriched, basal-like, and normal-like tumors, compared to Luminal tumors. WCHS data showed similar patterns, although the associations did not reach statistical significance.

Among methylation probes within *AKT1*, a gene that was relatively under-expressed in tumors from Black vs. White women, we observed two CpG island loci exhibiting significantly higher DNA methylation (β) values in Black than White women (cg07197515 and cg02884928) and one with a higher β value in White than Black women (cg15957959) (FDR corrected p < 0.05 for all three loci; Supplemental Fig. 1). The methylation levels of these loci were inversely associated with gene expression of *AKT1* (r = − 0.21, − 0.22, and − 0.29, respectively; Supplemental Fig. 2). A consistent finding on the correlation was observed in WCHS participants between methylation of cg02884928 and *AKT1* expression (r = − 0.24, p = 0.08; data not shown). One CpG island locus in *RPS6KB2* showed higher DNA methylation in Black women compared to White women (cg18905855, FDR corrected p-value = 7.65E−08; Supplemental Fig. 3). There was a weak positive correlation between methylation of cg18905855 and gene expression of *RPS6KB2* (r = 0.10; Supplemental Fig. 4).

## Discussion

In this study of breast tumors from Black and White women, race, grade, HER2 status, and molecular subtype were associated with expression levels of genes we examined in the mTOR pathway in breast tumors. *AKT1* and *RPS6KB2* were relatively under-expressed in tumors from Black women compared to White women, a finding that was in the opposite direction of our hypothesis.

AKT is one of the most activated proteins in breast cancer [31–33]. We observed differential gene expression of *AKT1* by HER2 status and stage among the study individuals and by intrinsic subtype and stage among the TCGA individuals. High vs. normal BMI is associated with higher levels of gene expression for AKT-mTOR pathway genes in breast cancer [26, 34–36], but in our analysis, the association of race and *AKT1* expression did not change after adjusting for BMI. We are unable to explore other factors (e.g., frequencies of mutations and circulating IGF levels), which may differ by race and potentially influence *AKT1* gene expression. Our post-hoc analysis showed some indications that differential DNA methylation might play a role in the relative under-expression of *AKT1* among Black women compared to White women. We demonstrated that two CpG probes in *AKT1* exhibited significantly higher methylation in Black patients compared to White patients and the methylation levels were inversely associated with gene expression levels.

Amplification and expression of *RPS6KB2* in breast cancer tissues are correlated with decreased tamoxifen responsiveness and poor prognosis in ER+/PR + tumors [11]. The resistance to endocrine therapy is likely an important contributor to the racial disparity in breast cancer mortality because it is clear that Black women with ER+/PR + tumors have higher mortality [6] as well as breast cancer recurrence [7], compared to White women with ER+/PR + tumors, and the difference is not observed among Black and White women with ER−/PR− tumors. However, we observed a relative under-expression of *RPS6KB2* in breast cancer overall and in luminal tumors from Black women compared to White women, a finding that was opposite to our hypothesis. The explanation is not clear. With regard to clinicopathological factors, we found that *RPS6KB2* was relatively over-expressed in higher grade and HER2 + tumors in our samples. In TCGA, *RPS6KB2* was relatively over-expressed in HER2-enriched and basal-like tumors, relative to luminal tumors, although these associations were not statistically significant. We found one CpG probe with differential DNA methylation in *RPS6KB2*, although the methylation levels were not inversely associated with the gene expression levels. These associations may require further examination by including information on endocrine therapy as well as more upstream and downstream factors of the mTOR pathway and DNA methylation.

Along with *RPS6KB2*, high expression of *EIF4EBP1* might be indicative of more aggressive breast tumor phenotypes as overexpression of *EIF4EBP1* is associated with poor breast cancer prognosis [37]. We observed an association between higher tumor grade and higher gene expression of *EIF4EBP1*. This finding is consistent with the literature reporting that p-4E-BP1 is expressed in poorly differentiated tumors and associated with high pathologic grade and poor breast cancer prognosis [38]. In TCGA, we observed that *EIF4EBP1* was relatively over-expressed in HER2-enriched and basal-like tumors. We observed the same patterns in our sample, although the associations did not reach statistical significance.

We observed associations of higher grade, HER2+, and triple-negative breast tumors with lower gene expression of *TSC1*, a result consistent with our analysis in TCGA for HER2-enriched and basal-like tumors. Invasive breast tumors have lower protein expression of TSC1 and TSC2 compared to the normal mammary epithelium [39]. *TSC1* is a tumor suppressor gene that encodes hamartin, which interacts with and stabilizes the GTPase activating protein, tuberin, encoded by *TSC2* gene. The hamartin-tuberin complex is a negative regulator of mTORC1 signaling [24]. Dysregulated TSC1/TSC2 complex results in the overactivation of mTOR signaling leading to cell proliferation [39]. Thus, lower expression of *TSC1*, along with higher expression of *RPS6KB2* and *EIF4EBP1*, can be indicative of more aggressive breast cancer phenotypes.

Observations from our data suggest that the patterns of tumor characteristics in relation to gene expression may not be consistent with the patterns of the phosphorylation of proteins through which the mTOR pathway responds to the extracellular environment, e.g., energy and amino acid influx. In our previous study using tissue microarray data from the WCHS participants, phosphoprotein expression of MTOR, AKT1, and S6K1 were consistently lower in patients with a higher grade tumor, larger tumor, more advanced disease, or TNBC [13]. However, in the current study, *MTOR* and *AKT1* gene expression was not associated with these tumor characteristics. For *RPS6KB2*, gene expression seemed to indicate more aggressive tumor characteristics. A limitation of this comparison was that *RPS6KB2* encodes protein S6K2, not S6K1 for which we had data on the phosphoprotein expression. Although S6K1 and S6K2 may have a similar function in the mTOR pathway, a more direct comparison between expression levels of *RPS6KB2* and p-S6K2 is needed. Also, we were unable to include tumor size in the analyses because the data were unavailable among the patients selected from PNSR although patients from WCHS had the variables. The exact reason for the discrepancies between gene and phosphoprotein expression in relation to tumor characteristics is unclear. A hypothesis is that compared to gene expression, protein phosphorylation may be more likely to be suppressed or lose its function in breast tumors with more aggressive features.

Limitations of this study should be noted. Our study only utilized data on a small panel of mTOR pathway genes. The results would inform a more comprehensive gene set analysis that would yield more robust results. Also, data on tumor grade was unavailable in the TCGA data. In the post hoc analysis, while methylation loci that impact gene expression tend to be located in gene promoter regions, we were unable to confirm whether the significant CpG loci found in our exploratory analysis of DNA methylation were in the promoter regions of the genes studied here. The Illumina Infinium Human Methylation 450k array probes were ambiguously mapped to the human genome [40], and the assay may be biased towards CGI and promoters [41]. Also, the loci of *AKT1* and *RPS6KB2* that were differentially methylated between tumors from Black women and White women were scattered throughout the genes. DNA methylation results should be interpreted with caution, and an in-depth DNA methylation analysis is warranted.

In conclusion, *AKT1* and *RPS6KB2*, two important prognostic predictors in the mTOR pathway, are expressed differently in breast tumors in Black and White women. Whether DNA methylation plays a role in Black-White gene expression differences specific to the mTOR pathway warrants further research. Race and clinicopathological variables of breast cancer should be considered in studying the prognosis and endocrine therapy resistance related to mTOR pathway genes. Our findings may inform more comprehensive investigations of gene expression in the mTOR pathway in relation to prognosis and treatment resistance overall and by race.

## Supplementary Information

Below is the link to the electronic supplementary material.

Supplementary material 1 (DOCX 553.5 kb)

## Data Availability

The data supporting the findings of this study are not publicly available to protect patient privacy. The data will be made available to authorized researchers with the approval of the Women’s Circle of Health Study (WCHS) committee and relevant Institutional Review Boards.
