# Supplementary material for: mTOR pathway gene expression in association with race and clinicopathological characteristics in Black and White breast cancer patients
Source: Discov Oncol. 2022 May 24;13:34. doi: 10.1007/s12672-022-00497-y (PMC9130392; doi:10.1007/s12672-022-00497-y)
Supplement: Supplementary file 1 — Supplementary material 1 (DOCX 553.5 kb) [file 12672_2022_497_MOESM1_ESM.docx]

**Supplemental Table 1:** Pearson Correlation Coefficients for mTOR Pathway Gene Expression levels^1,2^

| Overall |  |  |  |  |  |
| --- | --- | --- | --- | --- | --- |
|  | *AKT1* | *EIF4EBP1* | *MTOR* | *RPS6KB2* | *TSC1* |
| *AKT1* | 1.00 |  |  |  |  |
| *EIF4EBP1* | 0.07  (0.18) | 1.00 |  |  |  |
| *MTOR* | 0.17  (0.0008) | 0.08  (0.12) | 1.00 |  |  |
| *RPS6KB2* | 0.46  (<0.0001) | 0.30  (<0.0001) | 0.18  (0.0004) | 1.00 |  |
| *TSC1* | 0.19  (0.0003) | -0.42  (<0.0001) | 0.12  (0.0198) | -0.10  (0.0525) | 1.00 |
|  |  |  |  |  |  |
| White race |  |  |  |  |  |
| *AKT1* | 1.00 |  |  |  |  |
| *EIF4EBP1* | 0.09  (0.24) | 1.00 |  |  |  |
| *MTOR* | 0.14  (0.0701) | 0.16  (0.0313) | 1.00 |  |  |
| *RPS6KB2* | 0.43  (0.0001) | 0.35  (<0.0001) | 0.23  (0.0019) | 1.00 |  |
| *TSC1* | 0.22  (0.0036) | -0.34  (<0.0001) | 0.14  (0.0663) | -0.08  (0.2632) | 1.00 |
|  |  |  |  |  |  |
| Black race |  |  |  |  |  |
| *AKT1* | 1.00 |  |  |  |  |
| *EIF4EBP1* | 0.07  (0.3712) | 1.00 |  |  |  |
| *MTOR* | 0.21  (0.0035) | 0.01  (0.9030) | 1.00 |  |  |
| *RPS6KB2* | 0.47  (<0.0001) | 0.27  (0.0001) | 0.13  (0.0673) | 1.00 |  |
| *TSC1* | 0.16  (0.0299) | -0.49  (<0.0001) | 0.10  (0.1587) | -0.13  (0.0823) | 1.00 |

^1^mTOR pathway gene expression levels were log2 transformed values

^2^P-values in parentheses

Supplemental Table 2: Association between race and clinicopathological characteristics (including AJCC stage) and gene expression of the mTOR pathway in breast cancer (n= 321)^1^

|  | *AKT1* | | *EIF4EBP1* | | *MTOR* | | *RPS6KB2* | | *TSC1* | |
| --- | --- | --- | --- | --- | --- | --- | --- | --- | --- | --- |
| Characteristic | Log2  fold-change  (95% CI) | p-value | Log2  fold-change  (95% CI) | p-value | Log2  fold-change  (95% CI) | p-value | Log2  fold-change  (95% CI) | p-value | Log2  fold-change  (95% CI) | p-value |
| Race |  |  |  |  |  |  |  |  |  |  |
| White | Ref |  | Ref |  | ref |  | Ref |  | ref |  |
| Black | -0.30  (-0.43, -0.16) | **<0.0001** | 0.01  (-0.18, 0.20) | 0.9062 | -0.03  (-0.10, 0.04) | 0.3751 | -0.09  (-0.18, -0.004) | **0.0413** | -0.01  (-0.09, 0.06) | 0.7346 |
| Age |  |  |  |  |  |  |  |  |  |  |
| ≤40 | Ref |  | Ref |  | ref |  | Ref |  | ref |  |
| 41 - 50 | 0.06  (-0.17, 0.29) | 0.6049 | 0.14 (-0.18, 0.47) | 0.3929 | 0.02  (-0.09, 0.14) | 0.6928 | -0.07  (-0.22, 0.08) | 0.3664 | 0.09  (-0.03, 0.22) | 0.1359 |
| 51 - 65 | 0.02  (-0.19, 0.24) | 0.8418 | 0.14 (-0.16, 0.45) | 0.3654 | -0.01  (-0.12, 0.10) | 0.7969 | -0.12  (-0.26, 0.02) | 0.0970 | 0.05  (-0.07, 0.17) | 0.4095 |
| >65 | -0.06  (-0.31, 0.19) | 0.6463 | -0.07  (-0.43, 0.29) | 0.6935 | 0.05  (-0.08, 0.18) | 0.4535 | -0.15  (-0.32, 0.01) | 0.0652 | 0.12  (-0.02, 0.26) | 0.0859 |
| Tumor grade |  |  |  |  |  |  |  |  |  |  |
| Low | Ref |  | ref |  | ref |  | Ref |  | Ref |  |
| Intermediate | 0.20  (-0.08, 0.47) | 0.1620 | 0.38 (-0.01, 0.77) | 0.0558 | -0.04  (-0.18, 0.10) | 0.5960 | 0.17  (-0.003, 0.35) | 0.0543 | -0.01  (-0.16, 0.14) | 0.9007 |
| High | 0.12  (-0.16, 0.41) | 0.3865 | 0.79 (0.39, 1.19) | **0.0001** | -0.06  (-0.20, 0.08) | 0.4143 | 0.29  (0.11, 0.47) | **0.0016** | -0.20  (-0.35, -0.05) | **0.0097** |
| AJCC stage |  |  |  |  |  |  |  |  |  |  |
| Stage I | Ref |  | ref |  | ref |  | Ref |  | ref |  |
| Stage II | 0.10  (-0.05, 0.24) | 0.1954 | 0.25  (0.04, 0.46) | **0.0191** | 0.04  (-0.04, 0.11) | 0.3040 | 0.06  (-0.03, 0.16) | 0.1953 | -0.06  (-0.14, 0.02) | 0.1157 |
| Stage III | 0.35 (0.06, 0.64) | **0.0176** | 0.24  (-0.17, 0.64) | 0.2498 | 0.02  (-0.13, 0.16) | 0.8169 | 0.07  (-0.12, 0.25) | 0.4816 | -0.03  (-0.19, 0.13) | 0.7016 |
| Stage IV | 0.64  (-0.05, 1.33) | 0.0696 | 0.87  (-0.11, 1.85) | 0.0809 | 0.13  (-0.22, 0.48) | 0.4786 | 0.74  (0.30, 1.19) | **0.0011** | 0.15  (-0.22, 0.53) | 0.4175 |
| ER status |  |  |  |  |  |  |  |  |  |  |
| Positive | Ref |  | ref |  | ref |  | ref |  | ref |  |
| Negative | 0.02  (-0.58, 0.63) | 0.9369) | -0.08  (-0.94, 0.78) | 0.8536 | -0.03  (-0.33, 0.28) | 0.8628 | -0.03  (-0.42, 0.36) | 0.8665 | 0.06  (-0.27, 0.39) | 0.7097 |
| PR status |  |  |  |  |  |  |  |  |  |  |
| Positive | Ref |  | Ref |  | ref |  | ref |  | ref |  |
| Negative | 0.17  (-0.08, 0.42) | 0.1816 | 0.01  (-0.34, 0.36) | 0.9572 | -0.06  (-0.18, 0.07) | 0.3720 | 0.06  (-0.10, 0.23) | 0.4301 | 0.09  (-0.05, 0.22) | 0.2111 |
| HER2 status |  |  |  |  |  |  |  |  |  |  |
| Negative | Ref |  | Ref |  | ref |  | ref |  | ref |  |
| Positive | 0.19  (-0.01, 0.40) | 0.0614 | 0.10  (-0.18, 0.39) | 0.4858 | -0.08  (-0.19, 0.02) | 0.1047 | 0.13  (0.001, 0.26) | **0.0487** | -0.12  (-0.23, -0.01) | **0.0306** |
| Molecular subtype |  |  |  |  |  |  |  |  |  |  |
| Luminal | Ref |  | ref |  | ref |  | ref |  | ref |  |
| HER2+ | 0.12  (-0.57, 0.81) | 0.7272 | 0.35  (-0.62, 1.32) | 0.4811 | 0.17  (-0.18, 0.52) | 0.3388 | 0.03  (-0.41, 0.48) | 0.8785 | -0.24  (-0.61, 0.14) | 0.2125 |
| Triple-negative | -0.45  (-1.09, 0.20) | 0.1731 | 0.43  (-0.48, 1.34) | 0.3572 | 0.05  (-0.28, 0.37) | 0.7827 | -0.06  (-0.48, 0.35) | 0.7599 | -0.40  (-0.75, -0.05) | **0.0242** |

Abbreviations: CI, confidence interval

^1^Linear regression models included race, age, tumor grade, AJCC stage, ER status, PR status, HER2 status, and molecular subtype.

**Supplemental Table 3**: Association between race, age, and clinicopathological characteristics and gene expression of the mTOR pathway in breast cancer after additionally controlling for BMI (n=150)^1^

|  | *AKT1* | | *EIF4EBP1* | | *MTOR* | | *RPS6KB2* | | *TSC1* | |
| --- | --- | --- | --- | --- | --- | --- | --- | --- | --- | --- |
| Characteristic | Log2  fold-change  (95% CI) | p-value | Log2  fold-change  (95% CI) | p-value | Log2  fold-change  (95% CI) | p-value | Log2  fold-change  (95% CI) | p-value | Log2  fold-change  (95% CI) | p-value |
| Race |  |  |  |  |  |  |  |  |  |  |
| White | Ref |  | ref |  | ref |  | ref |  | ref |  |
| Black | -0.48  (-0.73, -0.23) | **0.0002** | 0.02  (-0.35, 0.40) | 0.8983 | -0.04  (-0.16, 0.08) | 0.5380 | -0.23  (-0.40, -0.06) | **0.0100** | -0.07  (-0.21, 0.07) | 0.3415 |
| Age |  |  |  |  |  |  |  |  |  |  |
| ≤40 | Ref |  | Ref |  | ref |  | ref |  | ref |  |
| 41 - 50 | 0.16  (-0.20, 0.53) | 0.3788 | -0.29  (-0.84, 0.26) | 0.3032 | -0.09  (-0.27, 0.09) | 0.3230 | -0.25  (-0.50, 0.002) | 0.0517 | 0.16  (-0.06, 0.37) | 0.1460 |
| 51 - 65 | 0.12  (-0.24, 0.49) | 0.5008 | -0.09  (-0.63, 0.45) | 0.7548 | -0.08  (-0.26, 0.09) | 0.3655 | -0.27  (-0.52, -0.02) | **0.0322** | 0.08  (-0.13, 0.28) | 0.4710 |
| >65 | -0.04  (-0.47, 0.40) | 0.8702 | -0.69  (-1.34, -0.04) | **0.0383** | -0.09  (-0.30, 0.12) | 0.3837 | -0.34  (-0.64, -0.04) | **0.0255** | 0.11  (-0.14, 0.36) | 0.3673 |
| BMI |  |  |  |  |  |  |  |  |  |  |
| <25 | Ref |  | ref |  | ref |  | ref |  | ref |  |
| ≥25 - <30 | -0.04  (-0.33, 0.24) | 0.7849 | -0.22  (-0.65, 0.21) | 0.3174 | 0.12  (-0.02, 0.26) | 0.1001 | -0.02  (-0.21, 0.18) | 0.8674 | -0.04  (-0.21, 0.12) | 0.6047 |
| ≥30 | 0.08  (-0.18, 0.34) | 0.5446 | -0.15  (-0.53, 0.23) | 0.4472 | -0.04  (-0.17, 0.08) | 0.4867 | -0.06  (-0.23, 0.12) | 0.5256 | 0.02  (-0.13, 0.16) | 0.8148 |
| Tumor grade |  |  |  |  |  |  |  |  |  |  |
| Low | Ref |  | ref |  | Ref |  | ref |  | ref |  |
| Intermediate | 0.20  (-0.18, 0.57) | 0.3040 | 0.41  (-0.15, 0.97) | 0.1481 | 0.03  (-0.15, 0.22) | 0.7077 | 0.23  (-0.03, 0.48) | 0.0811 | 0.01  (-0.21, 0.22) | 0.9436 |
| High | 0.22  (-0.17, 0.60) | 0.2699 | 0.81  (0.24, 1.38) | **0.0058** | -0.01  (-0.20, 0.17) | 0.8858 | 0.37  (0.11, 0.63) | **0.0056** | -0.10  (-0.32, 0.12) | 0.3819 |
| ER status |  |  |  |  |  |  |  |  |  |  |
| Positive | Ref |  | ref |  | Ref |  | ref |  | ref |  |
| Negative | -0.07  (-0.69, 0.54) | 0.8114 | 0.04  (-0.87, 0.95) | 0.9316 | -0.05  (-0.35, 0.24) | 0.7235 | -0.07  (-0.49, 0.35) | 0.7442 | 0.02  (-0.33, 0.37) | 0.9090 |
| PR status |  |  |  |  |  |  |  |  |  |  |
| Positive | Ref |  | ref |  | Ref |  | ref |  | ref |  |
| Negative | 0.17  (-0.21, 0.56) | 0.3687 | -0.26  (-0.83, 0.31) | 0.3630 | -0.15  (-0.34, 0.03) | 0.1013 | 0.05  (-0.21, 0.31) | 0.7103 | 0.01  (-0.21, 0.23) | 0.9120 |
| HER2 status |  |  |  |  |  |  |  |  |  |  |
| Negative | Ref |  | ref |  | Ref |  | ref |  | ref |  |
| Positive | 0.22  (-0.09, 0.53) | 0.1561 | 0.63  (0.17, 1.10) | **0.0074** | -0.02  (-0.17, 0.13) | 0.7530 | 0.30  (0.09, 0.51) | **0.0055** | -0.14  (-0.31, 0.04) | 0.1317 |
| Molecular subtype |  |  |  |  |  |  |  |  |  |  |
| Luminal | Ref |  | ref |  | Ref |  | ref |  | ref |  |
| HER2+ | -0.01  (-0.81, 0.79) | 0.9797 | 0.27  (-0.92, 1.47) | 0.6528 | 0.25  (-0.14, 0.64) | 0.2097 | -0.14  (-0.69, 0.41) | 0.6090 | -0.30  (-0.76, 0.16) | 0.1965 |
| Triple-negative | -0.30  (-1.02, 0.42) | 0.4140 | 0.75  (-0.33, 1.82) | 0.1715 | 0.19  (-0.16, 0.53) | 0.2916 | -0.01  (-0.50, 0.48) | 0.9592 | -0.35  (-0.76, 0.07) | 0.0996 |

Abbreviations: CI, confidence interval

^1^Linear regression models included race, age, tumor grade, ER status, PR status, HER2 status, molecular subtype, and BMI.

**Supplemental Table 4**: Characteristics of the study participants in TCGA breast cancer data (N=332)

| Characteristic | Black (n = 30) | White (n = 302) |
| --- | --- | --- |
|  | n (%) | n (%) |
| Age |  |  |
| ≤40 | 8 (26.67) | 32 (10.60) |
| 41 - 50 | 4 (13.33) | 62 (20.53) |
| 51 - 60 | 12 (40.00) | 141 (46.69) |
| >60 | 6 (20.00) | 67 (22.19) |
| ER status |  |  |
| Positive | 20 (66.67) | 234 (77.48) |
| Negative | 10 (33.33) | 68 (22.52) |
| PR status |  |  |
| Positive | 19 (63.33) | 197 (65.23) |
| Negative | 11 (36.67) | 105 (34.77) |
| HER2 status |  |  |
| Negative | 27 (90.00) | 262 (86.75) |
| Positive | 3 (10.00) | 40 (13.25) |
| PAM 50 |  |  |
| Luminal | 17 (56.67) | 213 (70.53) |
| HER2-enriched | 4 (13.33) | 21 (6.95) |
| Basal-like | 9 (30.00) | 62 (20.53) |
| Normal-like | 0 (0.00) | 6 (1.99) |
| AJCC stage |  |  |
| Stage I | 4 (13.33) | 35 (11.59) |
| Stage II | 17 (56.67) | 196 (64.90) |
| Stage III | 9 (30.00) | 64 (21.19) |
| Stage IV | 0 (0.00) | 7 (2.32) |

**Supplemental Table 5**. Gene expression (log2-transformed values) of the mTOR pathway in breast cancer according to race, age, and clinicopathological characteristics in TCGA breast cancer data (N=332)

| Characteristic |  | *AKT1* | | *EIF4EBP1* | | *MTOR* | | *RPS6KB2* | | *TSC1* | |
| --- | --- | --- | --- | --- | --- | --- | --- | --- | --- | --- | --- |
|  | n | Mean ± SD | p-value | Mean ± SD | p-value | Mean± SD | p-value | Mean± SD | p-value | Mean± SD | p-value |
| Race |  |  | 0.8806 |  | 0.9562 |  | 0.4345 |  | 0.9773 |  | 0.9474 |
| White | 300 | 18.38±0.57 |  | 19.55±1.18 |  | 17.25±0.45 |  | 17.12±0.70 |  | 16.41±0.49 |  |
| Black | 30 | 18.39±0.55 |  | 19.54±1.08 |  | 17.18±0.43 |  | 17.12±0.65 |  | 16.41±0.48 |  |
| Age |  |  | 0.8703 |  | 0.7996 |  | 0.2759 |  | 0.8320 |  | 0.2242 |
| ≤40 | 39 | 18.32±0.52 |  | 19.70±1.14 |  | 17.19±0.41 |  | 17.19±0.63 |  | 16.35±0.34 |  |
| 41 - 50 | 66 | 18.42±0.53 |  | 19.47±1.29 |  | 17.32±0.48 |  | 17.16±0.71 |  | 16.42±0.50 |  |
| 51 - 60 | 152 | 18.39±0.56 |  | 19.56±1.11 |  | 17.25±0.45 |  | 17.09±0.67 |  | 16.46±0.47 |  |
| >60 | 73 | 18.38±0.64 |  | 19.51±1.21 |  | 17.19±0.43 |  | 17.14±0.78 |  | 16.33±0.58 |  |
| ER status |  |  | 0.3173 |  | **<0.0001** |  | 0.4313 |  | **<0.0001** |  | **<0.0001** |
| Positive | 253 | 18.40±0.51 |  | 19.33±1.10 |  | 17.23±0.41 |  | 17.04±0.69 |  | 16.48±0.48 |  |
| Negative | 77 | 18.33±0.72 |  | 20.25± 1.12 |  | 17.28±0.56 |  | 17.41±0.65 |  | 16.18±0.46 |  |
| PR status |  |  | 0.4896 |  | **<0.0001** |  | 0.1883 |  | **<0.0001** |  | **<0.0001** |
| Positive | 215 | 18.37±0.49 |  | 19.25±1.13 |  | 17.22±0.40 |  | 17.00±0.70 |  | 16.51±0.47 |  |
| Negative | 115 | 18.41±0.69 |  | 20.09±1.03 |  | 17.29±0.52 |  | 17.35±0.63 |  | 16.23±0.48 |  |
| HER2 status |  |  | **0.0002** |  | 0.7505 |  | 0.9896 |  | **<0.0014** |  | 0.3323 |
| Negative | 287 | 18.34±0.54 |  | 19.56±1.18 |  | 17.24±0.45 |  | 17.08±0.69 |  | 16.42±0.48 |  |
| Positive | 43 | 18.69±0.63 |  | 19.49±1.07 |  | 17.24±0.45 |  | 17.44±0.66 |  | 16.34±0.55 |  |
| PAM 50 |  |  | **<0.0001** |  | **<0.0001** |  | 0.0820 |  | **<0.0001** |  | **<0.0001** |
| Luminal | 229 | 18.39±0.50 |  | 19.26±1.09 |  | 17.23±0.40 |  | 16.99±0.67 |  | 16.50±0.46 |  |
| HER2 enriched | 25 | 18.80±0.68 |  | 20.04±1.06 |  | 17.17±0.47 |  | 17.59±0.74 |  | 16.16±0.42 |  |
| Basal-like | 70 | 18.20±0.63 |  | 20.24±1.07 |  | 17.35±0.57 |  | 17.36±0.63 |  | 16.20±0.52 |  |
| Normal-like | 6 | 18.36±0.61 |  | 20.45±1.12 |  | 16.98±0.40 |  | 17.36±0.71 |  | 16.55±0.21 |  |
| AJCC stage |  |  | **0.0267** |  | **0.0035** |  | **0.0448** |  | 0.1514 |  | **0.0038** |
| Stage I | 38 | 18.15±0.53 |  | 19.06±0.82 |  | 17.43±0.30 |  | 16.88±0.63 |  | 16.61±0.45 |  |
| Stage II | 212 | 18.39±0.53 |  | 19.61±1.22 |  | 17.23±0.46 |  | 17.16±0.69 |  | 16.39±0.48 |  |
| Stage III | 73 | 18.49±0.65 |  | 19.52±1.06 |  | 17.18±0.44 |  | 17.14±0.75 |  | 16.43±0.49 |  |
| Stage IV | 7 | 18.34±0.61 |  | 20.64±1.20 |  | 17.26±0.58 |  | 17.23±0.60 |  | 15.94±0.60 |  |

Values are mean (standard variation) of gene expression levels.

**Supplemental Table 6**: Association between race and clinicopathological characteristics and gene expression of the mTOR pathway in TCGA breast cancer data^1^

| Characteristic | *AKT1* | | *EIF4EBP1* | | *MTOR* | | *RPS6KB2* | | *TSC1* | |
| --- | --- | --- | --- | --- | --- | --- | --- | --- | --- | --- |
|  | Log2  fold-change  (95% CI) | p-value | Log2  fold-change  (95% CI) | p-value | Log2  fold-change  (95% CI) | p-value | Log2  fold-change  (95% CI) | p-value | Log2  fold-change  (95% CI) | p-value |
| Race |  |  |  |  |  |  |  |  |  |  |
| White (0) | Ref |  | ref |  | Ref |  | ref |  | ref |  |
| Black (1) | 0.05  (-0.16, 0.26) | 0.6411 | -0.11  (-0.52, 0.30) | 0.6121 | -0.06  (-0.23, 0.11) | 0.5169 | -0.04  (-0.30, 0.21) | 0.7305 | 0.03  (-0.15, 0.21) | 0.7319 |
| Age |  |  |  |  |  |  |  |  |  |  |
| ≤40 | Ref |  | ref |  | Ref |  | ref |  | Ref |  |
| 41 - 50 | 0.14  (-0.09, 0.36) | 0.2305 | -0.18  (-0.62, 0.25) | 0.4028 | 0.15  (-0.03, 0.33) | 0.1132 | -0.001  (-0.27, 0.27) | 0.9930 | 0.04  (-0.15, 0.23) | 0.6570 |
| 51 - 60 | 0.09  (-0.11, 0.29) | 0.3614 | -0.03  (-0.41, 0.36) | 0.8892 | 0.05  (-0.11, 0.21) | 0.5258 | -0.03  (-0.27, 0.21) | 0.7967 | 0.07  (-0.10, 0.24) | 0.4025 |
| >60 | 0.09  (-0.13, 0.31) | 0.4295 | 0.05  (-0.38, 0.48) | 0.8136 | -0.01  (-0.18, 0.17) | 0.9481 | 0.08  (-0.18, 0.35) | 0.5366 | -0.10  (-0.29, 0.09) | 0.2895 |
| ER status |  |  |  |  |  |  |  |  |  |  |
| Positive | Ref |  | ref |  | ref |  | ref |  | Ref |  |
| Negative | -0.08  (-0.33, 0.16) | 0.5188 | 0.23  (-0.25, 0.71) | 0.3426 | -0.08  (-0.28, 0.11) | 0.4003 | 0.05  (-0.25, 0.34) | 0.7639 | -0.04  (-0.25, 0.16) | 0.6697 |
| PR status |  |  |  |  |  |  |  |  |  |  |
| Positive | Ref |  | ref |  | ref |  | ref |  | ref |  |
| Negative | 0.21  (0.03, 0.39) | **0.0232** | 0.35  (-0.01, 0.70) | 0.0575 | 0.06  (-0.09, 0.21) | 0.4033 | 0.14  (-0.08, 0.36) | 0.2191 | -0.12  (-0.27, 0.04) | 0.1428 |
| HER2 status |  |  |  |  |  |  |  |  |  |  |
| Negative | Ref |  | ref |  | ref |  | ref |  | ref |  |
| Positive | 0.16  (-0.05, 0.37) | 0.1354 | -0.26  (-0.67, 0.16) | 0.2224 | 0.07  (-0.10, 0.24) | 0.4238 | 0.26  (-0.002, 0.52) | 0.0519 | 0.02  (-0.16, 0.20) | 0.8211 |
| Intrinsic subtype (PAM 50) |  |  |  |  |  |  |  |  |  |  |
| Luminal | Ref |  | ref |  | ref |  | ref |  | ref |  |
| HER2-enriched | 0.22  (-0.07, 0.51) | 0.1294 | 0.70  (0.13, 1.27) | **0.0161** | -0.09  (-0.33, 0.14) | 0.4388 | 0.35  (-0.003, 0.71) | 0.0522 | -0.31  (-0.55, -0.06) | **0.0152** |
| Basal-like | -0.27  (-0.52, -0.03) | **0.0306** | 0.52  (0.03, 1.00) | **0.0361** | 0.14  (-0.06, 0.34) | 0.1819 | 0.26  (-0.04, 0.56) | 0.0906 | -0.19  (-0.40, 0.02) | 0.0795 |
| Normal like | -0.11  (-0.56, 0.33) | 0.6156 | 1.14  (0.26, 2.02) | **0.0111** | -0.28  (-0.65, 0.09) | 0.1341 | 0.36  (-0.19, 0.91) | 0.2026 | 0.04  (-0.34, 0.42) | 0.8323 |
| AJCC stage |  |  |  |  |  |  |  |  |  |  |
| Stage I | ref |  | ref |  | ref |  | ref |  | ref |  |
| Stage II | 0.22  (0.03, 0.41) | **0.0247** | 0.45  (0.08, 0.83) | **0.0177** | -0.21  (-0.36, -0.05) | **0.0089** | 0.20  (-0.04, 0.43) | 0.1013 | -0.19  (-0.35, -0.02) | **0.0242** |
| Stage III | 0.32  (0.10, 0.53) | **0.0046** | 0.45  (0.02, 0.88) | **0.0383** | -0.24  (-0.42, -0.06) | **0.0077** | 0.23  (-0.04, 0.50) | 0.0892 | -0.18  (-0.36, 0.01) | 0.0569 |
| Stage IV | 0.21  (-0.24, 0.65) | 0.3603 | 1.46  (0.59, 2.32) | **0.0010** | -0.19  (-0.55, 0.17) | 0.3105 | 0.31  (-0.23, 0.85) | 0.2613 | -0.62  (-1.00, 0.25) | **0.0012** |

^1^Linear regression models included for race, age, ER status, PR status, HER2 status, intrinsic subtype and, AJCC stage


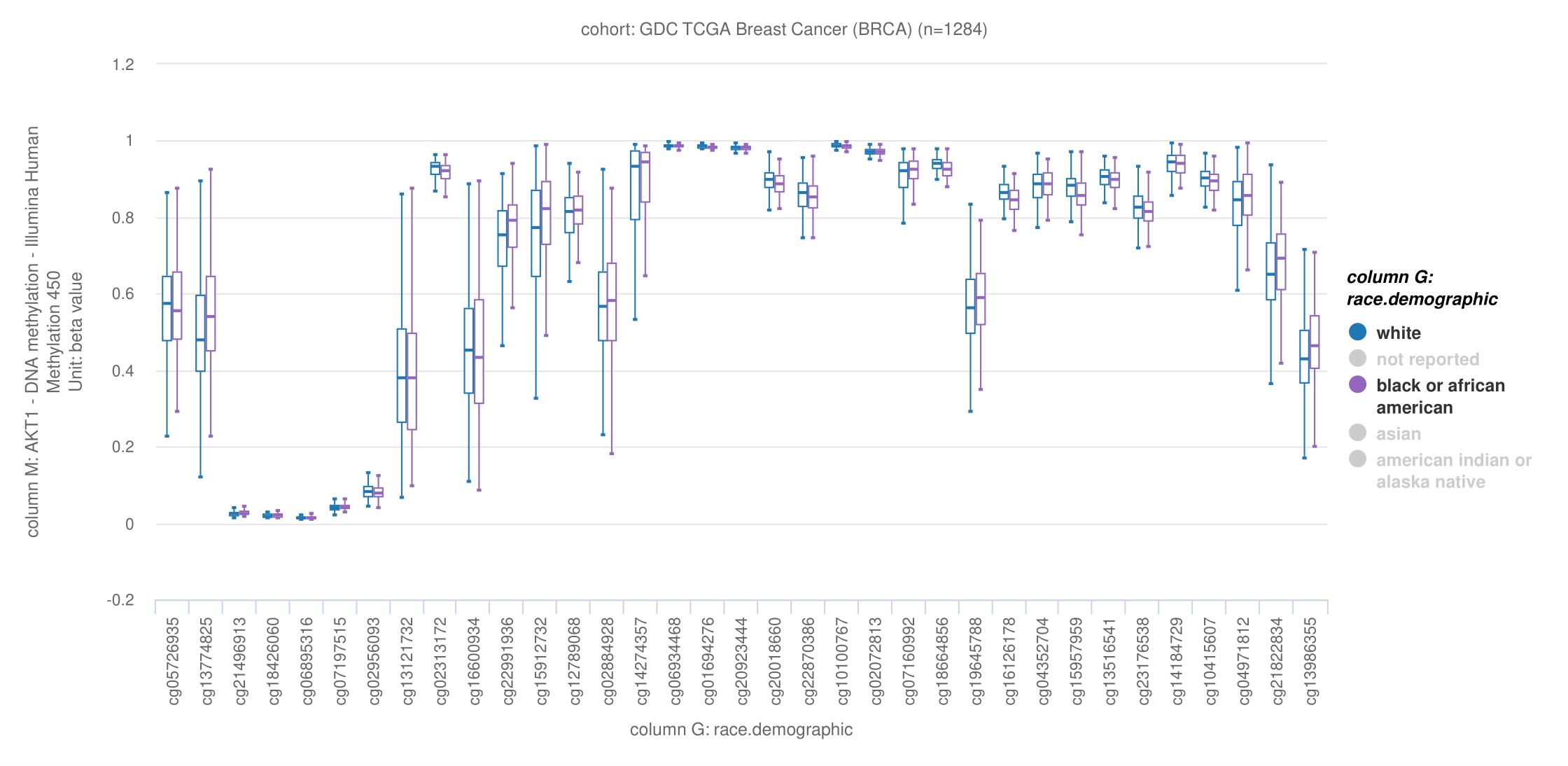


**Supplemental Figure 1**: CpG probes within *AKT1* that exhibited differential DNA methylation in Black race vs White race.

*Footnote showing probes and p-values.

| ***AKT1*** |  |  |  |  |  |
| --- | --- | --- | --- | --- | --- |
| **CpG Probe** | **p-value** | **q-value** | **Significant (q<0.05)** | **CpG Genomic location** | **Race with higher DNA methylation** |
| **Cg05726935** | **0.02616** | 0.070431 | 0 | S_Shore | White |
| **cg13774825** | **0.002757** | 0.024124 | 1 | S_Shore | Black |
| cg21496913 | 0.6513 | 0.670456 | 0 | Island |  |
| cg18426060 | 0.1601 | 0.24363 | 0 | Island |  |
| cg06895316 | 0.7765 | 0.7765 | 0 | Island |  |
| **cg07197515** | **0.004865** | 0.034055 | 1 | Island | Black |
| cg02956093 | 0.2434 | 0.331019 | 0 | Island |  |
| cg13121732 | 0.2095 | 0.305521 | 0 | N_Shore |  |
| **cg02313172** | **0.0005132** | 0.011982 | 1 | N_Shore | White |
| cg16600934 | 0.07984 | 0.133067 | 0 | N_Shelf |  |
| cg22991936 | 0.05587 | 0.110211 | 0 | S_Shelf |  |
| **cg15912732** | **0.02439** | 0.070431 | 0 | S_Shelf | Black |
| cg12789068 | 0.6320 | 0.670456 | 0 | S_Shelf |  |
| **cg02884928** | **0.01398** | 0.04893 | 1 | Island | Black |
| cg14274357 | 0.3397 | 0.407517 | 0 | Island |  |
| cg06934468 | 0.07615 | 0.133067 | 0 | N_Shelf |  |
| **cg01694276** | **0.001027** | 0.011982 | 1 | NA | White |
| **cg20923444** | **0.0009568** | 0.011982 | 1 | NA | White |
| **cg20018660** | **0.006483** | 0.037818 | 1 | NA | White |
| cg22870386 | 0.6322 | 0.670456 | 0 | NA |  |
| cg10100767 | 0.3229 | 0.403625 | 0 | NA |  |
| **cg02072813** | **0.04632** | 0.10808 | 0 | NA | Black |
| cg07160992 | 0.05668 | 0.110211 | 0 | S_Shelf |  |
| **cg18664856** | **0.02929** | 0.073225 | 0 | Island | White |
| cg19645788 | 0.05470 | 0.110211 | 0 | Island |  |
| **cg16126178** | **0.009455** | 0.037831 | 1 | NA | White |
| cg04352704 | 0.6479 | 0.670456 | 0 | Island |  |
| **cg15957959** | **0.009728** | 0.037831 | 1 | Island | White |
| cg13516541 | 0.2590 | 0.335741 | 0 | Island |  |
| cg23176538 | 0.1022 | 0.162591 | 0 | N_Shore |  |
| cg14184729 | 0.2459 | 0.331019 | 0 | N_Shore |  |
| cg10415607 | 0.3493 | 0.407517 | 0 | N_Shore |  |
| cg04971812 | 0.07712 | 0.133067 | 0 | N_Shelf |  |
| **cg21822834** | **0.008520** | 0.037831 | 1 | N_Shelf | Black |
| **cg13986355** | **0.01659** | 0.052786 | 0 | N_Shelf | Black |


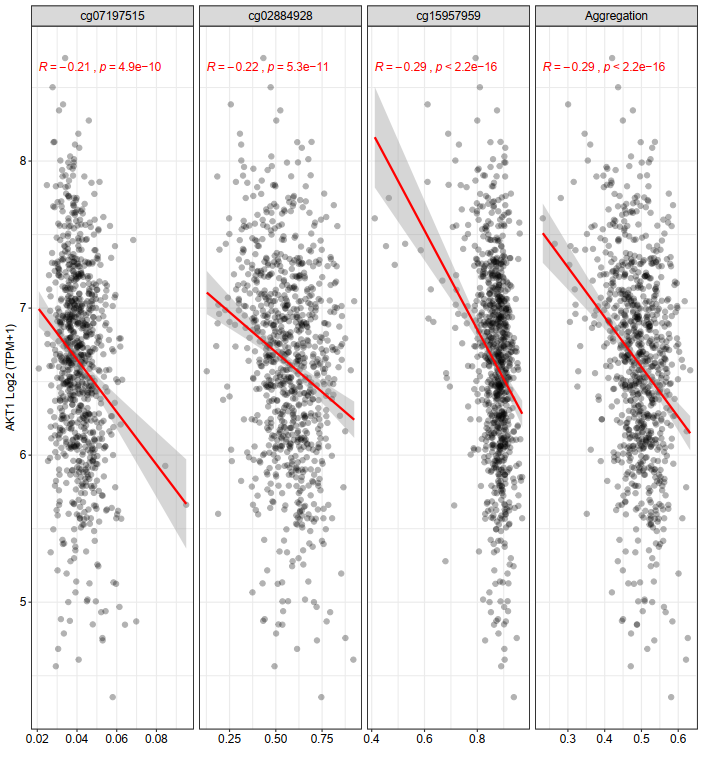


**Supplemental Figure 2**: Gene-level correlation analysis between gene expression of *AKT1* and the methylation of CpG probes.


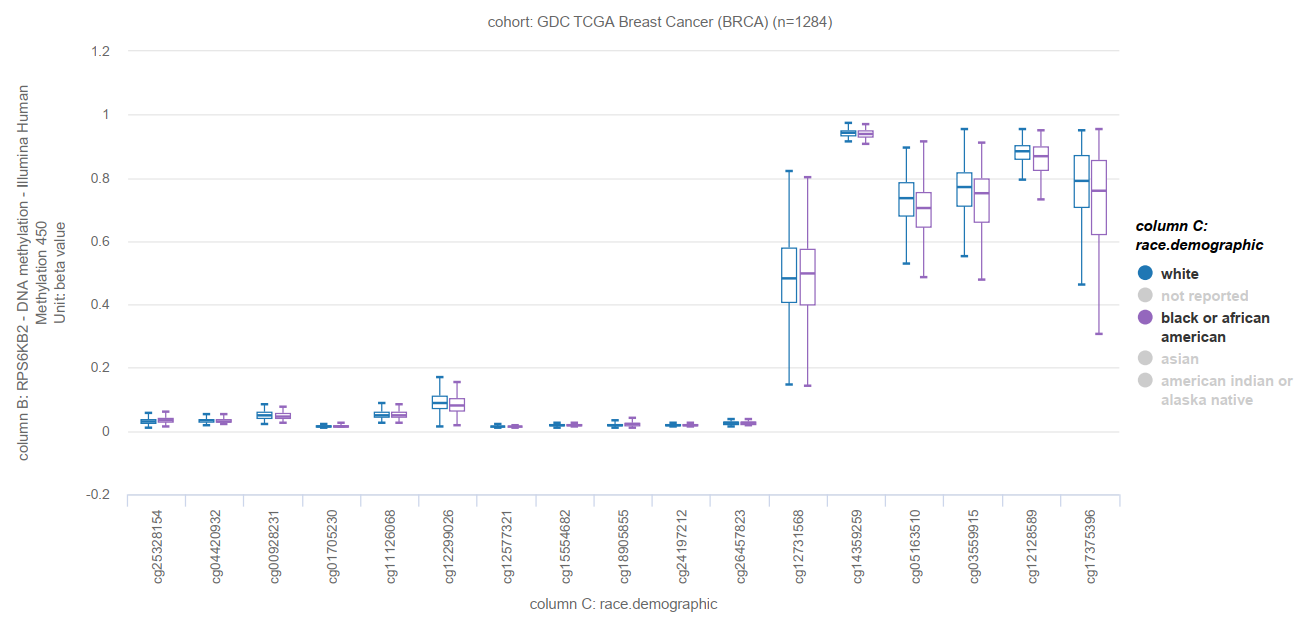


**Supplemental Figure 3**: CpG probes within *RPS6KB2* that exhibited differential DNA methylation in Black race vs White race.

*Footnote showing probes and p-values

| ***RPS6KB2*** |  |  |  |  |  |
| --- | --- | --- | --- | --- | --- |
| **CpG Probe** | **p-value** | **q-value** | **Significant (q<0.05)** | **CpG Genomic Location** | **Race with higher DNA methylation** |
| Cg25328154 | 0.9622 | 0.9622 | 0 | N_Shore |  |
| cg04420932 | 0.8459 | 0.898769 | 0 | N_Shore |  |
| cg00928231 | 0.2467 | 0.465989 | 0 | N_Shore |  |
| cg01705230 | 0.07698 | 0.186951 | 0 | Island |  |
| cg11126068 | 0.3352 | 0.518036 | 0 | Island |  |
| cg12299026 | 0.1687 | 0.358488 | 0 | Island |  |
| cg12577321 | 0.6830 | 0.774067 | 0 | Island |  |
| cg15554682 | 0.3332 | 0.518036 | 0 | Island |  |
| **cg18905855** | **4.501e-9** | 7.65E-08 | 1 | Island | Black |
| cg24197212 | 0.6515 | 0.774067 | 0 | Island |  |
| cg26457823 | 0.5950 | 0.774067 | 0 | S_Shore |  |
| cg12731568 | 0.6821 | 0.774067 | 0 | S_Shore |  |
| **cg14359259** | **0.0001262** | 0.000536 | 1 | N_Shelf | White |
| **cg05163510** | **0.002800** | 0.009466 | 1 | N_Shelf | White |
| **cg03559915** | **0.00006065** | 0.000344 | 1 | N_Shelf | White |
| **cg12128589** | **0.000003219** | 2.74E-05 | 1 | N_Shore | White |
| **cg17375396** | **0.003341** | 0.009466 | 1 | N_Shore | White |


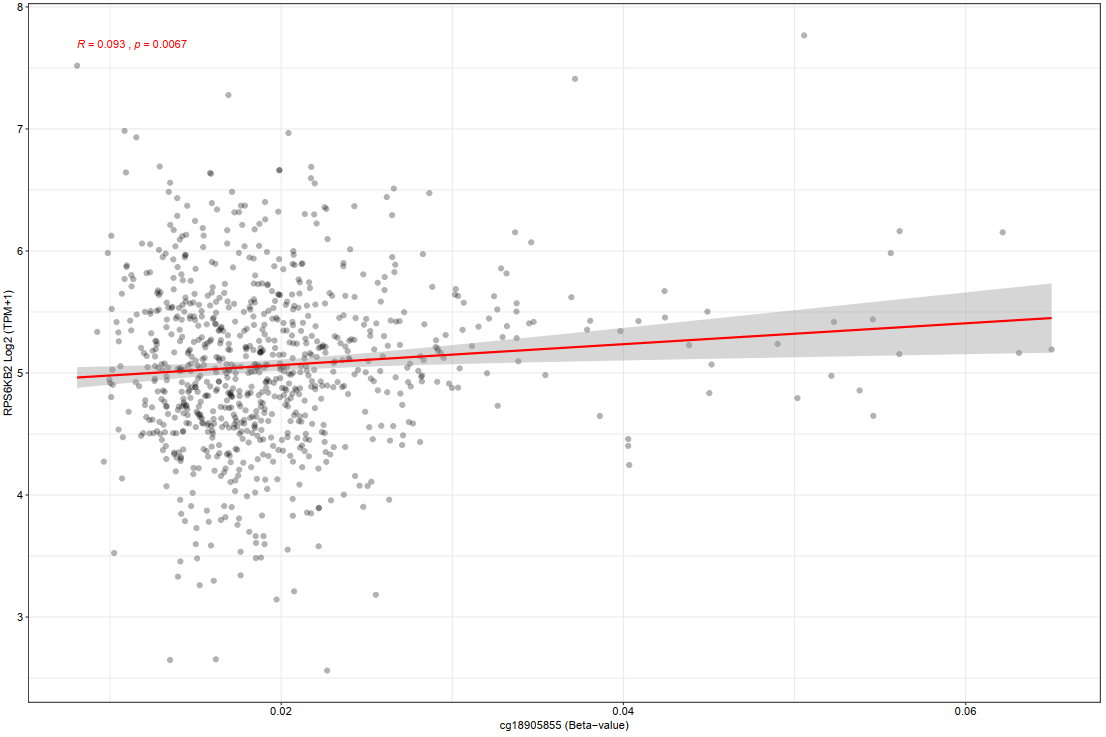


**Supplemental Figure 4**: Gene-level correlation analysis between gene expression of *RPS6KB2* and the methylation of CpG probe.
